# Impact of isolation and hospitalisation for people with TB

**DOI:** 10.5588/ijtldopen.26.0100

**Published:** 2026-08-11

**Authors:** I. Vavliakis, M.T. Brummelaar, H.A.M. Kerstjens, K. Kroon, M.S. Bolhuis, O.W. Akkerman

**Affiliations:** 1 Department of Pulmonary Diseases and Tuberculosis, University Medical Center Groningen, University of Groningen, Groningen, the Netherlands;; 2 Department of Οrthopedagogy, Faculty of Behaviour and Social Sciences, University of Groningen, Groningen, the Netherlands;; 3 University Medical Center Groningen, TB Center Beatrixoord, University of Groningen, Groningen, the Netherlands;; 4 Department of Clinical Pharmacy and Pharmacology, University Medical Center Groningen, University of Groningen, Groningen, the Netherlands.

**Keywords:** tuberculosis, person-centred care, emotional distress, social disruption, psychological support, stigma in health care

Dear Editor,

TB remains a major global health challenge, with 10.8 million cases and 1.3 million deaths in 2023.^1^ Low- and middle-income countries bear the greatest burden, where poverty, malnutrition, HIV/AIDS, multidrug-resistant TB and extensively drug-resistant TB, insufficient funding, and weak health systems impede progress.^1^ High-incidence countries use broad infection prevention and control (IPC) measures, including early TB detection^2,3^; whereas low-incidence countries (like the Netherlands) screen high-risk populations and use respiratory isolation (RI) to prevent transmission.^4–6^ Across all settings, context-appropriate IPC measures including RI protect vulnerable populations.^7,8^ However, little is known about the emotional and social effects of RI on people with TB (PwTB), as research has focused mainly on clinical outcomes. This study explores the emotional and social impacts of RI and hospitalisation, directly capturing PwTB perspectives to improve patient-centred care and inpatient isolation practices.

Ten adults with infectious pulmonary TB at TB Center Beatrixoord, University Medical Center Groningen, consented to participate in this mono-centre, prospective mixed-method study. People with extra-pulmonary TB were excluded as they are typically non-infectious and do not require RI. Participants were enrolled between 1 July 2022 and 22 June 2023. Participant characteristics are detailed in Table. A comprehensive protocol addressed informed consent, data management, interview training, confidentiality, and on-site aftercare. Semi-structured interviews explored emotional and social effects, with self-rated health on a 0–10 scale and translation provided when needed.^9^ Inductive and deductive thematic analysis identified key themes. The Hospital Anxiety and Depression Scale (HADS) assessed mental health,^10^ alongside demographic data, isolation duration, social and professional support, and satisfaction, summarised with descriptive statistics.^10^ Ethical clearance was waived (METC 2023-097).

**Table.** Baseline characteristics of participants.

Characteristics	Participant group (n = 10)
Gender	
Male/Female	8/2
Age (median + range)	40 (16–58)
WHO region	
European region	3
African region	4
South-East Asia region	2
Eastern Mediterranean region	1

The table presents the demographic and regional distribution of a participant group (n = 10). The ‘Male/Female’ column shows the gender ratio, while ‘Age (median + range)’ indicates the median age and range. The ‘WHO region’ specifies the regional classification of participants according to WHO criteria.

Thematic analysis of the 10 participants’ experiences revealed seven key themes. The theme ‘Symptoms and Health Trajectories’ highlighted the variety of symptoms, including fever, cough, night sweats, tinnitus, and body aches. Participants also rated their health on a 0–10 scale (0 = worst, 10 = best), with ratings from 5 to 8, indicating moderate to good self-perceived health despite substantial symptom burden and variability in illness trajectories. The theme ‘Support System’ showed the important role of family, friends, and self-reliance. One participant reported family conflicts due to her isolation, although she found comfort in her friend’s support. Another participant emphasised self-reliance as their main support, with minimal social contact apart from help from a friend offering accommodation. The theme ‘Information and Knowledge’ exposed disparities in participants’ understanding of TB and isolation. While some felt well-informed, others reported arriving without prior knowledge of their condition or the facility’s rules, indicating a need for improved communication on admission. The theme ‘Communication and Interaction’ reflected experiences with health care staff. Some participants reported language barriers that hindered building reciprocal relationships, while others highlighted positive, supportive interactions with staff: ‘My relationship with the staff has been positive; they are friendly and caring’. The theme ‘Isolation Experience’ detailed the emotional toll of long-term isolation. Participants described monotonous daily routines consisting of meals, short walks, and occasional leisure activities. While some struggled with solitude, others coped by maintaining regular contact with loved ones and focusing on small activities to stay engaged: ‘My days were quite monotonous… I tried to stay engaged, but the isolation was challenging’. The theme ‘Resilience and Motivation’ captured participants’ determination to improve their lives despite TB and isolation challenges. Many recommended engaging in brain-stimulating activities such as quizzes and games to maintain mental well-being during isolation periods. The theme ‘Stigma and Community Perception’ underscored the social stigma associated with TB. One participant reflected on community fear and negative reactions upon their diagnosis, highlighting the need for greater public awareness and empathy.

Quantitative data from the HADS showed that depression scores ranged from 3 to 12, with a mean of 5.7 and a standard deviation (SD) of 2.61 (Figure).^10^ Most participants fell within the normal range (0–7), although one (participant 10) had an abnormal score of 12. Anxiety scores varied from 1 to 11, with a mean of 5.9 and an SD of 2.98 (Figure). Participants 2 and 10 displayed elevated anxiety, scoring 11 (abnormal) and 9 (borderline abnormal), respectively. Full score distributions are shown in the Figure. When elevated HADS scores were identified, the treating physician was informed.

**Figure.**
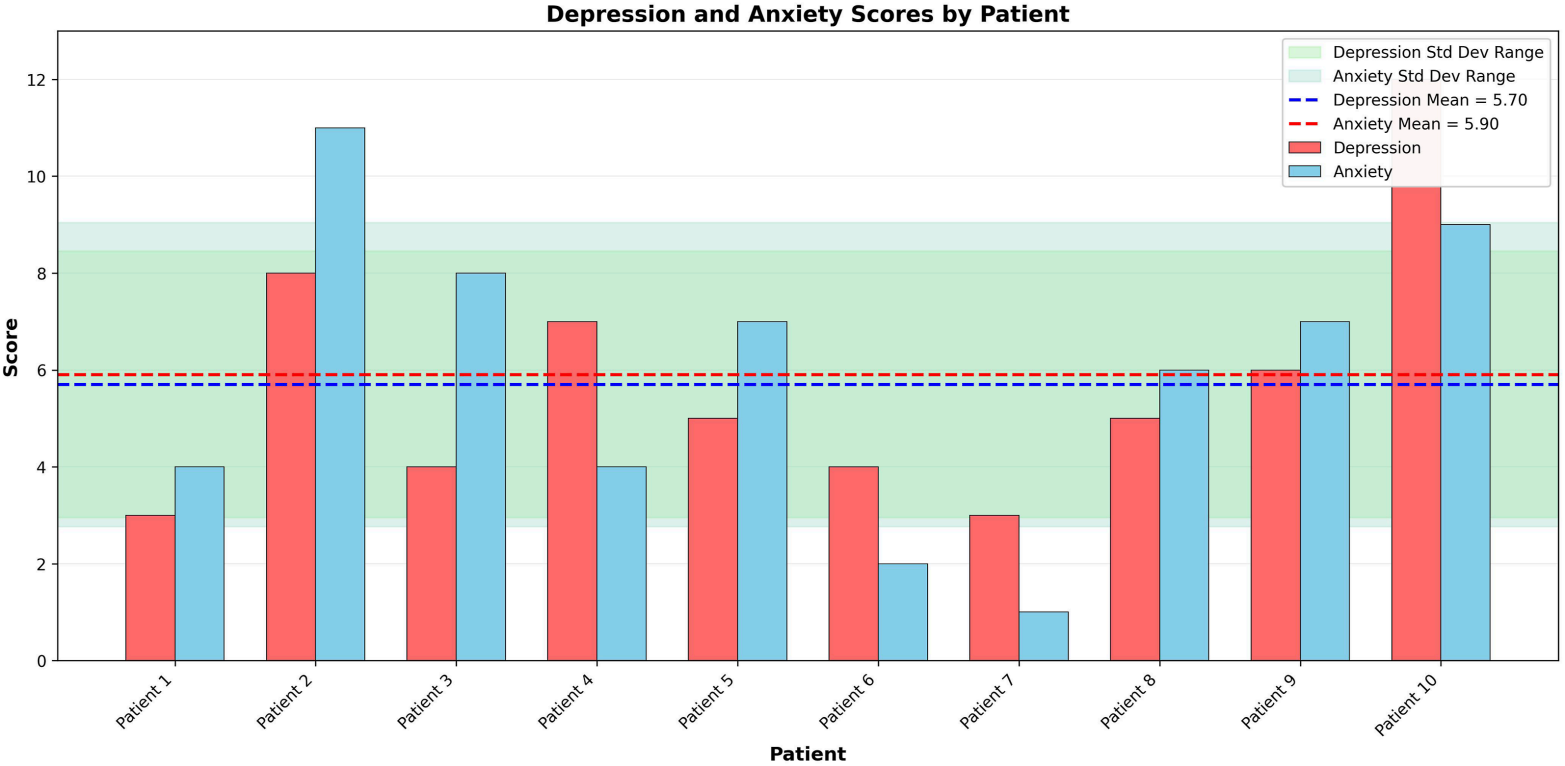
Combined depression and anxiety scores of participants. Depression (red) and anxiety (light blue) scores are shown for each participant based on the Hospital Anxiety and Depression Scale. Dashed lines mark the mean scores (depression: 5.7; anxiety: 5.9), with shaded areas indicating standard deviation ranges. Most participants scored within normal limits, though two showed borderline or abnormal levels, particularly participant 10, who reported the highest depression and elevated anxiety.

In this preliminary mixed-method study, we describe the physical, emotional, and social impacts of RI among PwTB in a low-incidence country. Qualitative and quantitative findings revealed substantial challenges affecting mental health, social connections, and quality of life. Key recommendations include integrating psychological support, improving staff communication, strengthening support systems, and incorporating engagement activities into isolation protocols to promote well-being and treatment adherence.

Thematic analysis revealed persistent symptoms such as cough, fever, and night sweats that disrupted daily life. Socially, participants reported marital conflicts and loneliness, with some relying on friends for support. These findings echo those of Juliet Addo et al.,^11^ who identified similar themes in Brazil, Russia, India, China, and South Africa, including economic hardship, stigma, family disruptions, and COVID-19 impacts. Such shared struggles highlight universal challenges faced by PwTB in isolation and the need for interventions that address both socio-economic and psychosocial dimensions. Our results also align with Mainga’s study in Zambia,^12^ which highlighted mental distress among PwTB, especially due to poverty-related stresses. While our study emphasises the emotional toll of isolation, Mainga’s work deepens the understanding of mental health challenges, reinforcing the call for integrated mental health services in TB care.^12^ Similarly, Oliva Rapoport’s study in Lima, Peru,^13^ found that prolonged isolation caused educational setbacks and emotional trauma among adolescent PwTB. Both studies advocate for re-evaluating isolation practices to align with evidence on TB transmission and human rights principles.^12,13^ Collectively, these global studies emphasise the importance of holistic TB care that addresses social and economic vulnerabilities and mental health, while ensuring that isolation practices remain evidence-based and rights-respecting to improve outcomes for PwTB.

Quantitative data from the HADS offered further insights into participants’ psychological states.^10^ Although most participants scored within the normal range, two displayed borderline abnormal or abnormal anxiety and/or depression levels, consistent with reports of psychological distress during isolation in other infectious diseases.^14^ Importantly, these symptoms cannot be attributed to isolation alone; they may reflect an interaction between isolation and pre-existing mental health, prior (potentially traumatic) experiences, and concurrent social or illness-related stressors. Taken together, the findings support a context-sensitive approach in which PwTB are routinely screened and offered timely, individualised psychosocial support during isolation.

A strength of this study is the mixed-method design, with in-depth interviews complemented by structured measures (HADS), enabling triangulation. Limitations relate mainly to transferability: recruitment from a single TB centre in a low-incidence setting limits generalisability. Interviews were also conducted at a single illness phase, so perspectives may evolve over time. Future work should include multiple sites, longitudinal follow-up, and perspectives of family members and health care professionals to contextualise patient experiences and guide practice changes.

In conclusion, this study highlights the complex effects of isolation on PwTB and the importance of patient-centred, holistic care approaches. Strengthening support systems, enhancing communication, and combating stigma are essential steps toward improving patient experiences and outcomes.
